# Structural alterations in early auditory cortex in borderline personality disorder with auditory verbal hallucinations

**DOI:** 10.1007/s00429-026-03162-0

**Published:** 2026-08-29

**Authors:** Yéléna Le Prieult, Marie-Luise Otte, Mike M. Schmitgen, Mert Koc, Chantal Tech, Robert Christian Wolf

**Affiliations:** 1 Department of General Psychiatry, Center for Psychosocial Medicine, Heidelberg University, Vossstrasse 4, Heidelberg, 69115 Germany; 2 University Hospital for Psychiatry and Psychotherapy III, Ulm University, Leimgrubenweg 12-14, Ulm, 89075 Germany; 3 German Center for Mental Health (DZPG), partner site Mannheim/ Heidelberg/Ulm (ZIHUb), Germany

**Keywords:** Auditory verbal hallucinations, Borderline personality disorder, MRI, voxel-based morphometry, surface-based morphometry

## Abstract

Auditory Verbal Hallucinations (AVH) are not exclusive to schizophrenia but also occur in other psychiatric disorders, including Borderline Personality Disorder (BPD). While AVH in SZ has been extensively studied, their neurobiological underpinnings in BPD remain poorly understood. Preliminary evidence suggests structural anomalies in auditory regions among BPD patients experiencing AVH, indicating potential neuroanatomical vulnerabilities. This study examined structural brain differences in female BPD patients with (BPD-AVH *n* = 20) and without (BPD-nAVH *n* = 27) a lifetime history of AVH, as well as healthy controls (HC, *n* = 30), using high-resolution structural MRI. Cortical thickness (CTh), gyrification (CG), and gray matter volume (GMV) were assessed in frontotemporal regions implicated in auditory and speech processing, including the inferior frontal gyrus and superior, middle, and transverse temporal gyri. Region-of-interest (ROI) analyses were performed via CAT12, with supplementary segmentation of Heschl’s gyrus using the TASH toolbox. Group comparisons were conducted using SPM12. Compared to both HC and BPD-nAVH, BPD-AVH patients showed significantly reduced CG in the right transverse temporal gyrus (rTTG), a region encompassing early auditory cortex. No significant group differences emerged in other ROI analyses, for GMV, CG or CTh. Reduced gyrification in the rTTG suggests aberrant cortical folding in early auditory regions among BPD patients with AVH. This may reflect disrupted neurodevelopmental processes that impair auditory signal encoding and internal vs. external sound source discrimination. These findings highlight the role of primary auditory cortex abnormalities in AVH and support transdiagnostic models emphasizing early sensory processing deficits.

**Supplementary Information:**

The online version contains supplementary material available at https://doi.org/10.1007/s00429-026-03162-0.

## Introduction

Borderline Personality Disorder (BPD) is a severe mental disorder marked by interpersonal hypersensitivity, unstable self-image, intense emotionality, and impulsive, often self-harming behaviors (Gunderson et al. 2018). In addition, transient psychotic symptoms, including paranoid ideation and auditory verbal hallucinations (AVH), are common, affecting 25–30% of outpatients and up to 50% of more severely ill individuals, complicating diagnosis and treatment (Niemantsverdriet et al. 2017). In BPD, lifetime prevalence of AVH ranges from 22 to 50% (Niemantsverdriet et al. 2017; Slotema et al. 2012). Such symptoms are often threatening or degrading (Beatson 2019) and associated with poor outcomes, including elevated suicide risk and diminished quality of life (Slotema et al. 2012; Thomas et al. 2007). AVH in BPD have been long debated regarding their phenomenological status. Earlier frameworks described these experiences as “pseudo-psychotic” or as “pseudo-hallucinations” but this distinction has been increasingly questioned. Recent research highlights substantial phenomenological overlap with hallucinations in psychotic disorders (Niemantsverdriet et al. 2017; Schroeder et al. 2013; Tseng and Georgiades 2024). AVH are transdiagnostic phenomena observed across various severe mental disorders, including major depression and schizophrenia (Kay et al. 1987). Over the past two decades, extensive neuroscientific research has investigated their underlying neural mechanisms, with the majority of studies focusing on individuals with SZ. In these patients, AVH have been linked to structural and functional abnormalities in regions central to language and auditory processing, including the superior temporal gyrus (STG), with AVH severity being associated with GMV reductions in the left STG (Allen et al. 2008; Hugdahl 2015). In addition, findings of transverse temporal gyrus (TTG) and planum temporale (PT) alterations implicate impairment of early auditory processing in AVH (Dierks et al. 1999). Moreover, patients with SZ and AVH frequently show structural abnormalities in the inferior frontal gyrus (IFG) (Barber et al. 2021; Romeo and Spironelli 2022; Ysbæk-Nielsen et al. 2024). The middle temporal gyrus (MTG) has also emerged as critical: cortical thinning in the left MTG is observed in SZ patients with AVH, suggesting deficits in auditory-language integration (Cui et al. 2018). Eventually, prior work has linked GMV reductions in the MTG to AVH severity (Cui et al. 2018; Modinos et al. 2013).

Despite increasing clinical recognition of AVH in BPD, their neurobiological basis remains far less studied than in SZ. Neuroimaging studies of AVH in SZ consistently implicate frontotemporal networks involved in auditory perception and language processing, including the superior temporal cortex and IFG (Allen et al. 2008; Modinos et al. 2013). In contrast, neuroimaging research in BPD has mainly focused on affective dysregulation and frontolimbic circuitry (Ruocco et al. 2012; Schulze et al. 2016), with only a small number of studies specifically examining the neural correlates of AVH in BPD. These limited investigations suggest involvement of similar frontotemporal regions (Niemantsverdriet et al. 2017; Slotema et al. 2012), but overall evidence remains scarce. Consequently, it remains unclear whether AVH in BPD share neural substrates with psychotic disorders. In contrast to the extensive research on AVH in SZ, the neural correlates of these symptoms in BPD remain poorly understood, despite their high prevalence and clinical significance in this population. This gap in knowledge concerns even the fundamental question of whether, and if so which, core regions of the language and auditory pathways are involved in AVH in BPD. For instance, while the involvement of IFG/Broca’s and STG/Wernicke’s areas and early auditory cortex areas is well established in AVH models of SZ, their role in BPD remains unclear. In a previous pilot study, surface-based morphometry was used to investigate cortical structural differences in female BPD patients with a lifetime history of AVH, compared to healthy controls (HC) and BPD patients without AVH (non-AVH). The structural findings indicated abnormalities in temporal regions, including reduced cortical thickness and increased gyrification in the STG. Notably, BPD patients with AVH (BPD-AVH) also showed abnormal folding in the TTG compared to BPD- patients without AHV (BPD-nAVH). These preliminary findings suggest that structural deviations in early auditory regions, particularly the TTG, may contribute to vulnerability to AVH in BPD (Kubera et al. 2023). Sulcal morphology is a stable marker of early neurodevelopment linked to psychosis and hallucination risk. These features form prenatally/early postnatally and persist throughout life (Mangin et al. 2010; Zilles et al. 2013). Recent studies have linked atypical sulcal patterns in language/auditory-related regions to AVH. For example, a study (Salgado-Pineda et al. 2025) reported that sulcal pit morphology in Broca’s area distinguished SZ patients who experienced AVH from those who did not, supporting a neurodevelopmental contribution to auditory verbal perceptual anomalies. Thus, sulcal morphology complements surface-based metrics like CG as neurodevelopmentally anchored marker of AVH.

In this study, we expand on previous research (Kubera et al. 2023) by employing structural MRI alongside both voxel- and surface-based morphometry to investigate GMV, cortical thickness (CTh), and cortical gyrification (CG) in regions implicated in AVH, as indicated by transdiagnostic research. The regions of interest (ROI) included the IFG (triangular, orbital, and opercular parts), STG, MTG, PT, and TTG. AVH are thought to arise from dysfunctions in a frontotemporal network that supports auditory perception, speech processing and self-monitoring. The STG/TTG is central to early auditory encoding: alterations here are linked to AVH across diagnoses (Allen et al. 2008; Modinos et al. 2013). PT show abnormal lateralization and stream segregation in hallucinating individuals (Hugdahl et al. 2009; Shapleske et al. 1999). MTG abnormalities affect semantic processing, shaping the linguistic content of hallucinations (Binder et al. 2009; Kühn and Gallinat 2012). IFG mediates speech production and source monitoring, potentially misattributing internal verbal material to external sources (Allen et al. 2006; Frith 1992a, b). Together, dysfunctions across these nodes seem to interact to produce AVH through deficits in sensory encoding, speech representation and cognitive control, with each node’s contribution varying among clinical groups. Additionally, we conducted a detailed morphometric analysis of the Heschl’s gyrus (HG), assessing volume, surface area, average and standard thickness, curvature and folding index and Gaussian and mean curvature to better elucidate its role in AVH. Utilizing TASH (Dalboni da Rocha et al. 2020) allowed for a more precise segmentation of HG, which is critical given the complex folding patterns and anatomical variability of these early auditory regions. This refined approach improves the accuracy of morphometric measurements and aids in detecting subtle structural differences that may underline AVH vulnerability. In line with other studies on AVH in SZ (Gaser et al. 2004; Palaniyappan et al. 2011), we predicted that BPD-AVH would exhibit distinct reduction of structural measures in these language and auditory network regions compared to BPD-nAVH patients and HC. Finally, we explored putative associations between brain morphology and clinical symptoms, both in terms of overall BPD symptom load and in terms of AVH-specific measures.

## Materials and methods

### Participants and psychometric instruments

This study was part of a larger project supported by the German Research Foundation (DFG) aiming at investigating neuronal correlates of AVH in BPD. To reduce clinical and phenotypic heterogeneity that has been frequently associated with gender (Bozzatello et al. 2024; Sanchious et al. 2024), only female participants were included in the study. BPD and HC participants were recruited at the Department of General Psychiatry at Heidelberg University, Germany and through advertisements on posters, flyers and social media platforms. All BPD patients were recruited according to the DSM-5 criteria and following further inclusion criteria i.e. right handed female participants, age between 18 and 65 years, stable psychotropic drug treatment for at least 2 weeks, no diagnosis of SZ, schizoaffective disorder or affective disorders with psychotic symptoms and patients with current use of psychotropic drugs fulfilling DSM-5 criteria for moderate substance use disorder (except nicotine) within three months prior to study enrollment, detailed lifetime history of substance use or abuse was not systematically collected. BPD-AVH patients had to present a history of AVH independent of symptom onset, duration or specific features of AVH and BPD-nAVH patients no life-time history of AVH. For all patients, the following exclusion criteria were applied: current and life-time neurological disease or current medical condition known to affect brain function, acute suicidal ideation and acute (self)aggressiveness and patients where drug or alcohol abuse did precede the first experience of AVH.

Psychometric instruments used in this study included the Borderline Symptom List 23 (Bohus et al. 2009), the Psychotic Symptom Rating Scale (Morrison 1998), the Hamilton Depression Scale (Hamilton 1960), the Childhood Trauma Questionnaire (Bernstein et al. 2003).

In the BPD-nAVH group, 22 patients received antidepressants, 10 patients received antipsychotics, three patient received anticonvulsants and one patient received psychostimulants. In the BPD-AVH group, 11 patients received antidepressants, eight patients received antipsychotics, three patients received anticonvulsants and two received psychostimulants (see supplementary table S1). Current comorbid mental disorders were present in 96.3% BPD-nAVH and in 95% BPD-AVH, predominantly major depressive disorder (MDD, BPD-nAVH: 88.5%, BPD-AVH: 84.2%) and post-traumatic stress disorder (PTSD, BPD-nAVH: 19.2%, BPD-AVH: 57.9%). Current anxiety disorder was present in 11.5% BPD-nAVH and in 10.5% BPD-AVH.

Thirty participants were included as HC after ruling out a history of mental disorders using the Mini International Neuropsychiatric Interview (Sheehan et al. 1998).

### Neuroimaging data acquisition

Structural imaging was performed using a 3 Tesla MRI Prisma System (Siemens, Erlangen) in the Neuroradiology Department of Heidelberg University Hospital. Among the sequences acquired, T1-weighted 3D magnetization-prepared rapid gradient echo (MP-RAGE) sequences were recorded with the following parameters: TR = 1900 ms, TE = 2.26 ms, flip angle = 9°, slice thickness = 1 mm, slice gap (distance factor) = 0.5 mm, field of view (FoV) = 256 mm and voxel size 1 × 1 × 1 mm.

### MRI preprocessing

Structural data preprocessing was performed using the Statistical Parametric Mapping package (SPM12, 2023, v.7771: http://www.fil.ion.ucl.ac.uk/spm/software/spm12 last accessed: June 18, 2025) in conjunction with the Computational Anatomy Toolbox for SPM (CAT12 12.7 (r2577): http://www.neuro.uni-jena.de/cat/ last accessed: June 30, 2025). SPM12 has been used to manually reorient the origin of the images to the anterior commissure. The structural T1-weighted images were preprocessed using the CAT12 toolbox (version 12.7, https://neuro-jena.github.io/cat/ last accessed: June 30, 2025) (Gaser et al. 2024), implemented in SPM12. The preprocessing included bias field correction to reduce intensity inhomogeneities, affine registration to MNI space and high-dimensional DARTEL normalization. Images were segmented into gray matter (Schmaal et al. 2017), white matter and cerebrospinal fluid. CTh and CG were estimated using the Projection-Based Thickness method (Dahnke et al. 2013). Surface reconstruction included topology correction and spherical mapping. All preprocessing steps were performed using CAT12 default parameters (Gaussian kernel of 8 mm FWHM for GMV, 15 mm for CTh and 20 mm for CG). T1-weighted images were evaluated using the quality assessment pipeline implemented in CAT12, which computes a weighted image quality rating (IQR) based on noise-to-contrast ratio, inhomogeneity-to-contrast ratio and spatial resolution (Gaser et al. 2024). Scans are assigned a categorical grade ranging from A + to F (excellent to unacceptable, respectively). We excluded all participants with an IQR of D- or worse (barely sufficient) which led to the exclusion of two BPD-nAVH participants and one HC, while all remaining scans were retained. Homogeneity in each group and between groups was checked as well. Total Intracranial Volume (Gaser et al.) was extracted for use as a covariate in the GMV-based 2nd level models. As a last step in CAT12, we extracted the data of the GMV, CTh and CG per ROI (i.e. right and left STG, MTG, PT, TTG and opercular, triangular and orbital part of IFG). The Neuromorphometrics and the Desikian-Killiany atlas, in CAT12, were used for ROI definition and data extraction in voxel- (GMV) and surface based (CTh and CG) data, respectively (see supplemental figure S1 for an overview of the used ROI-masks and supplemental figure S2 for an overview of the rTTG). Extracted ROI values were statistically analyzed offline using R (v.2023.06.0, https://www.R-project.org/ last access: June 30, 2025).

### HG segmentation

For HG-segmentation, T1-weighted anatomical MRI scans were, first, preprocessed using the FreeSurfer software suite (v. 7.4.1, https://surfer.nmr.mgh.harvard.edu/fswiki/ReleaseNotes last accessed: June 20, 2025) installed on an Ubuntu 22.04 LTS system and subsequently analyzed with TASH toolbox (Toolbox for Automated Segmentation of HG, https://www.db-thueringen.de/receive/dbt_mods_00020059 last accessed: June 17, 2025.) (Dalboni da Rocha et al. 2020). TASH is a specialized neuroimaging toolbox designed for the automated and detailed anatomical segmentation of HG from structural T1-weighted MRI scans (see supplementary materials for a detailed processing pipeline. In FreeSurfer, the data was preprocessed in a “tcsh” shell environment to ensure compatibility with the TASH scripts. The anatomical processing pipeline was carried out using FreeSurfer’s “recon-all” command to perform cortical reconstruction and volumetric segmentation. This included skull stripping, Talairach transformation, intensity normalization, tessellation, surface inflation and anatomical labeling.

TASH utilizes surface-based segmentation methods that leverage FreeSurfer cortical reconstructions to isolate HG from surrounding cortical areas. It applies a series of morphological and geometric operations on the cortical surface, such as erosion, dilation, thresholding and clustering, to delineate HG subregions and produce detailed anatomical measurements, including volume, surface area, average and standard thickness, Gaussian and mean curvature and curvature and folding index. The toolbox also integrates visualization steps to generate screenshots of the segmentation for quality control purposes, which led to exclusion of two BPD-nAVH, one BPD-AVH and one HC participants for the further analyses.

### Statistical analysis

All statistical analyses were conducted in R (v.2023.06.0, https://www.R-project.org/ last access: June 30, 2025) using the following packages: car (for ANCOVA type III), WRS2 (for robust ANCOVA), emmeans (for post-hoc comparisons), psych and stats (for correlation analyses, normality and homogeneity testing), corrplot (for correlation matrix visualization), ggplot2 and ggpubr (for data visualization and annotation) and tidyverse (for data management and preprocessing).

####  Region of interest analysis

A ROI analysis was chosen over a whole-brain approach to increase statistical sensitivity and reduce the risk of type I errors associated with multiple comparisons across the entire cortex (Poldrack 2007). The chosen ROIs were MTG, TTG, STG, PT, triangular, opercular and orbital part of IFG, left and right hemisphere separately. Given the strong a priori hypotheses regarding specific auditory and language-related regions as neural substrates of AVH, focusing on anatomically defined ROIs allowed for a targeted investigation aligned with existing neurobiological models (Allen et al. 2008; Modinos et al. 2013). For each dependent variable, i.e. GMV, CTh and CG, data was first tested for normality using the Shapiro-Wilk test. To evaluate the assumption of homogeneity of variances across groups, Levene’s test was performed. When both assumptions were met, an Analysis of Covariance (ANCOVA) was conducted, with group as the independent variable and covariates including medication, age, TIV (for GMV only) and Total Cortical Area (TCA, for area of the HG analysis only). If the homogeneity and normality assumptions were not met, robust ANCOVA was performed using the same covariates as above. Post hoc pairwise comparisons were conducted using Tukey’s Honestly Significant Difference (HSD) test for standard ANCOVA or a robust post hoc test for robust ANCOVA. Statistical significance was set at *p* < 0.05. To control for depressive symptom severity, a common feature in BPD and linked to cortical morphology, we added HAMD-21 scores as a covariate. ANCOVA type III and robust ANCOVA were rerun on CG measures with age, medication and HAMD-21 included, testing whether AVH related effects persist after accounting for depression. Additionally, as exploratory analyses, the main ANCOVA models were repeated including CTQ total scores, PTSD diagnosis and both CTQ total scores and PTSD diagnosis as additional covariates. Finally, FDR correction was applied for group comparison, meaning across all ANCOVAs test for all ROIs and structural measures (CG, CTh and GMV).

####  Medication effect

In all analyses, medication effects were accounted for as follows: antipsychotic and antidepressant medication were standardized as olanzapine equivalents (OLZe, (Leucht et al. 2015) and fluoxetine equivalent (FLUe, (Hayasaka et al. 2015), respectively. OLZe and FLUe were z**-**transformed to normalize the data, combined into a single composite variable “Medication” and added as covariate in subsequent analyses (see table S2). This approach ensures comparability across different substances and controls for potential confounding effects of pharmacological treatment.

####  Correlations between structural data and psychometric scores

Associations between structural ROI data (MTG, TTG, STG, PT, triangular, opercular and orbital part of IFG both hemispheres separately) and psychometric scores were examined in R. Prior to the correlation analyses, all structural and questionnaire data were separately tested for normality and homogeneity. Assumptions of homogeneity and normality being violated, Spearman’s rank correlation was applied for correlations. Correlation coefficients were calculated between extracted values of the ROIs and the HAMD-21, the BSL-23, CTQ and the PSYRATS sub scores. PSYRATS analyses were restricted to the hallucinations scale, and only the emotional, cognitive, and physical dimensions were included. For the PSYRATS Auditory Hallucinations subscale (Valmaggia et al. 2005; Woodward et al. 2014) (11 items), items were grouped into physical, emotional and cognitive domains. Demographic group differences were tested after assessing normality and homogeneity of variance. Comparisons between two groups were assessed using a student’s t-test when assumptions were held, otherwise Mann-Whitney U test was used. A Kruskal Wallis test was used for three group comparisons when data did not meet the normality assumption.

## Results

### Demographics and psychometric scores

Twenty-seven patients did not report AVH, neither lifetime nor current (BPD-nAVH), 20 reported a lifetime AVH (BPD-AVH). No significant age differences across groups were found (*p* = 0.24). Marginally significant differences in HAMD-21 and BSL-23 scores were detected between the patient groups (both HAMD-21 and BSL-23 *p* = 0.05), whereas CTQ scores significantly differed between BPD-AVH and BPD-nAVH (*p* = 0.04). Table 1 provides detailed demographics and psychometric scores and distribution of CTQ scores across groups can be observed in Supplementary Fig. 3 (see supplementary Material).

###  Structural MRI

For this study, data from 77 right-handed participants were included in the ROI analysis, comprising BPD patients without AVH (BPD-nAVH, *n* = 27), BPD patients with AVH (BPD-AVH, *n* = 20), and healthy controls (HC, *n* = 30). For the HG analysis via TASH (Dalboni da Rocha et al. 2020), 73 participants were included (BPD-nAVH, *n* = 25; BPD-AVH, *n* = 19; HC, *n* = 29). Exclusions consisted of two BPD-nAVH and one HC from the ROI analysis, and two BPD-nAVH, one BPD-AVH, and one HC from the HG analysis.

#### Between-group differences in GMV

No significant differences in BPD-AVH compared to BPD-nAVH and HC emerged in these analyses (see supplementary material).

#### Between-group differences in CTh

ROI-based analysis of CTh did not indicate differences in BPD-AVH compared to BPD-nAVH and HC (see supplementary material).

#### Between-group differences in CG

**Fig. 1:**
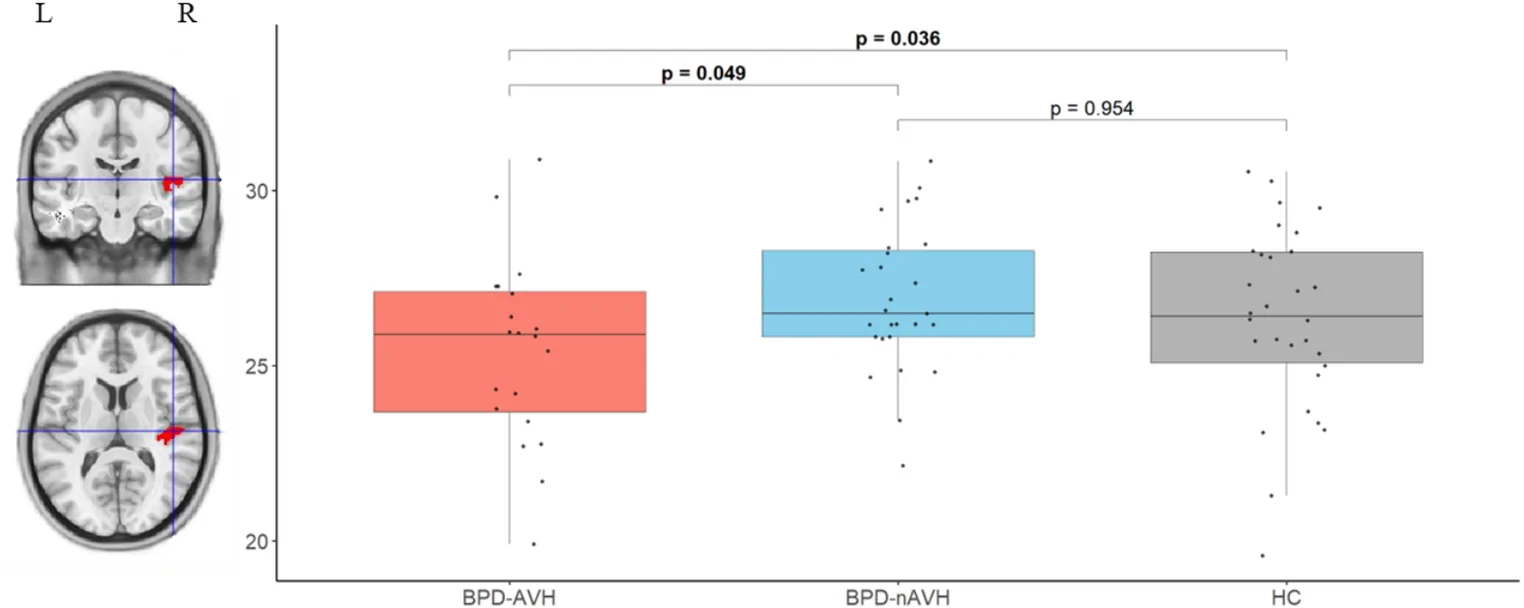
Cortical gyrification differences between patients with and without AVH and healthy controls. Left: visualization of the right transverse temporal gyrus. Mask created with MarsBaR (https://marsbar-toolbox.github.io/download.html last accessed: September 14, 2025) and displayed as an overlay onto the “Template_1mm.nii” template implemented in CAT12. This figure was created with SPM12 (https://github.com/spm/spm12 last accessed: September 14, 2025). Right: boxplot of the comparison of CG in the rTTG between groups. P-values of the ANCOVA type III test. Abbreviations: BPD-nAVH, borderline personality disorder without auditory verbal hallucinations; BPD-AVH, borderline personality disorder with auditory verbal hallucinations; HC, healthy controls

ANCOVA revealed a significant effect of group in the right TTG (rTTG; F (2, 72) = 389.59, *p* = 0.025). Post-hoc test revealed a reduced gyrification in BPD-AVH compared to both BPD-nAVH (*p* = 0.049) and HC (*p* = 0.036), see Fig. 1 and figure S4). Exploratory ANCOVA, including HAMD-21 produced results consistent with the primary analysis. Reduced right TTG CG remained significant (F (2, 71) = 382.69, *p* = 0.005). Post-hoc tests showed lower CG in BPD-AVH versus BPD-nAVH (*p* = 0.011) and HC (*p* = 0.008). The exploratory analyses including CTQ total scores, PTSD diagnosis, or both variables as additional covariates are reported in the Supplementary Material. In these analyses, the previously observed CG difference in the right TTG was no longer significant, whereas a significant CG difference emerged in the left opercular part of IFG when CTQ only and both PTSD and CTQ were considered as covariates. None of the observed effects survived FDR correction across all tested ROIs and structural metrics. An analysis of CG without medication as covariate showed no significant p-values after reperforming ANCOVA and robust ANCOVA tests.

#### Between-group differences following HG-segmentation

None of the metrics obtained by TASH showed significant differences in BPD-AVH compared to BPD-nAVH and HC.

#### Correlations between clinical parameters and rTTG CG

Spearman correlations showed no significant associations between CG in rTTG and PSYRATS-scores in BPD-AVH only ((PSYRATS mean score, p-value = 0.57, ρ = 0.13; PSYRATS physical sub score, p-value = 0.68, ρ = 0.1; PSYRATS emotional sub score, p-value = 0.68, ρ = 0.1; PSYRATS cognitive sub score, p-value = 0.36, ρ = 0.21) as well as with CTQ (p-value = 0.41, ρ = -0.19), BSL23 (p-value = 0.90, ρ = -0.03) and HAMD-21 questionnaires (p-value = 0.39, ρ = 0.2). No correlations survived FDR correction (all *p* > 0.05).

#### Exploratory trauma-related correlates of AVH phenomenology

In the BPD-AVH group, AVH severity did not differ significantly between patients with and without a PTSD diagnosis (Wilcoxon rank-sum test: W = 37.5, *p* = 0.36). In addition, in the BPD-AVH group, no significant association was observed between AVH severity and childhood trauma exposure, as measured by CTQ total scores (Pearson correlation, *r* = -0.16, *p* = 0.50).

Comparing BPD with and without AVH, a Pearson’s Chi-squared test showed a significant difference in PTSD between groups, with higher rates observed in the BPD-AVH group compared to the BPD-nAVH group (χ² (1) = 5.28, *p* = 0.022). In contrast, the incidence of MDD and anxiety disorders did not differ significantly between the patient groups.

## Discussion

In this study, we investigated structural brain alterations in frontotemporal regions implicated in auditory and language processing to elucidate neuronal correlates of AVH in BPD. Specifically, we investigated GMV, CTh and CG differences in key regions including the opercular, triangular and orbital parts of the IFG, STG, MTG, PT and TTG. Our results indicated significant reduction in CG in the right TTG in BPD-AVH compared to both HC and BPD-nAVH, while other regions did not show significant differences.

The TTG constitutes the PAC and serves as the first cortical relay for auditory information received from the thalamus (Morosan et al. 2001; Warrier et al. 2009). Structural features of the TTG, such as gyrification and CTh, are closely associated with the functional integrity of auditory perception (Morosan et al. 2001; Warrier et al. 2009). Decreased CG in this region may indicate atypical neurodevelopmental folding patterns or disruptions in early auditory integration capacity. In SZ, several studies have reported TTG abnormalities in individuals experiencing AVH, including reduced GMV (Modinos et al. 2013) and altered cortical morphology (Hubl et al. 2004). Although evidence in BPD remains scarce, our findings suggest that similar auditory processing abnormalities may underlie hallucinatory phenomena in this disorder, albeit with potentially different developmental trajectories or etiological contributions.

CG reduction confined to the right TTG with no differences observed in frontal regions or other temporal areas, potentially points to early auditory cortical architecture as a key factor in BPD with AVH. The lack of structural changes in inferior frontal or higher order temporal areas does not entirely rule out their involvement; Allen et al. (2008) and Cui et al. (2018) have linked these regions to source monitoring deficits and network abnormalities in hallucination. Thus, sensory level auditory alterations are a prominent structural vulnerability marker, while higher order linguistic or executive contributions may be subtler or distributed beyond ROI sensitivity. Although the present findings highlight a localized reduction of CG in the right TTG, they should not be interpreted as evidence for a singular or primary sensory deficit underlying AVH in BPD. CG is widely regarded as a marker of early neurodevelopmental processes and remains relatively stable across the lifespan (White et al. 2010; Zilles et al. 2013). Consequently, the observed alteration may reflect a neurodevelopmental vulnerability rather than a direct causal mechanism. Such vulnerability may interact with later environmental and clinical factors, e.g. particularly stress exposure, affective dysregulation and childhood trauma, all of which are strongly implicated in AVH across diagnostic categories (Laroi et al. 2012; Read et al. 2005). Salgado Pineda et al. (2025) linked sulcal pit abnormalities in speech related frontal areas to AVH in SZ, underscoring atypical folding as a vulnerability marker. Although our study focused on BPD rather than SZ, the convergence of findings across diagnostic categories might point to shared neurodevelopmental substrates rooted in early auditory/speech architecture: PAC specific gyrification changes suggest a sensory level contribution in BPD distinct from frontotemporal language alterations typical of SZ. This would align with developmental models of AVH that propose subtle abnormalities in the auditory cortex as foundational risk factors, particularly when combined with heightened emotional reactivity or impaired cognitive control mechanisms that are characteristic of BPD (Skåtun et al. 2016).

Despite the localized nature of our findings, they hold important implications for transdiagnostic models of AVH. The TTG is a core hub in auditory-linguistic networks and plays a crucial role in the integration of bottom-up sensory input with top-down cognitive expectations (Jasmin et al. 2019). Disruptions in its structure could impair the fidelity of auditory signal processing, making internally generated verbal thoughts or inner speech more susceptible to misperception as external voices. Our data does not provide direct support for inner-speech misattribution as a primary mechanism in BPD, despite its prominence in SZ models that implicate higher-order language and source-monitoring regions (inferior frontal, temporoparietal) (Allen et al. 2006; Frith 1992a, b). Stress-related deficits in cognitive control can worsen hallucinations even without lasting structural abnormalities (Waters et al. 2012). Thus, inner-speech misattribution might act as an interacting downstream process rather than the main explanatory mechanism in our study. The absence of significant alterations in frontal or temporal association cortices in our study highlights the need to consider disorder-specific structural signatures of AVH.

Importantly, affective dysregulation remains a hallmark of BPD and may contribute to the phenomenology of AVH. While we did not include core limbic regions typically implicated in emotional processing and no structural alterations were observed in the fronto-temporal regions examined here that are also linked to affective functions, the observed gyrification differences in the right TTG suggest that alterations in auditory cortical architecture may be relevant to AVH in BPD. Future studies integrating measures of emotional processing and auditory symptom characteristics are needed to clarify potential interactions between these domains. A detailed morphometric analysis of HG revealed no significant group differences in volume, surface area, CTh, mean curvature or local folding index, with CG in TTG emerging as the sole structural feature showing a significant alteration in BPD patients with AVH. In our study, the TTG encompasses PAC and adjacent auditory regions, whereas HG includes both primary and non-primary auditory areas and exhibits substantial interindividual variability (Hackett 2011; Morosan et al. 2001; Rademacher et al. 1993). Because anatomical boundaries do not map perfectly onto cytoarchitectonic fields, the observed TTG alterations cannot be attributed specifically to the primary auditory cortex. Nonetheless, the confinement of CG alterations to TTG, but not the broader HG, suggests involvement of early auditory cortical architecture. This pattern supports models proposing that AVH arise from aberrant early-stage auditory encoding rather than higher-order regions (Ford and Mathalon 2012; Jardri et al. 2011).

The CG’s involvement in rTTG supports a cortical anomaly underlying AVH in BPD rather than widespread pathology, consistent with prior links between CG abnormalities and perceptual disturbances in SZ spectrum disorders (Palaniyappan et al. 2013). Exploratory ANCOVA with HAMD-21 as covariate showed that the right TTG CG reduction persisted and even slightly strengthened. Depression typically involves frontolimbic/cingulate regions (Schmaal et al. 2017; Wise et al. 2017), whereas TTG, the core of PAC, is linked to AVH and early sensory abnormalities across disorders (Allen et al. 2008; Modinos et al. 2013). Thus, the effect’s stability after adjusting for depression suggests that the observed CG alteration is not solely attributable to depressive symptomatology but may reflect structural mechanisms more specifically associated with AVH rather than non-specific marker of depression in BPD. Importantly, the absence of correlations between rTTG CG and CTQ scores suggests that the observed structural alterations are unlikely to reflect direct neuroanatomical consequences of postnatal adverse experiences as captured by retrospective self-report. Instead, these findings should be considered as reflecting prenatal neurodevelopmental deviations associated with vulnerability to AVH. CTQ scores were nevertheless higher in patients with BPD and AVH than in patients with BPD without AVH, suggesting that childhood trauma may contribute to the clinical expression and severity of hallucinatory experiences even if it is not directly associated with the CG alterations observed here. This is consistent with a growing body of literature demonstrating elevated trauma burden in individuals with BPD who report psychotic-like experiences, including AVH (Niemantsverdriet et al. 2017; Slotema et al. 2012; Varese et al. 2012). Importantly, the absence of a structure-trauma correlation should not be interpreted as evidence against the role of trauma in AVH in BPD. Because CG is largely established prenatally, whereas CTQ reflects postnatal adverse experiences, our findings therefore suggest that AVH risk in BPD may be shaped by early neurodevelopmental vulnerability at the cortical level, with postnatal trauma acting as a precipitating or amplifying factor. This is consistent with stress-vulnerability models, in which adverse childhood experience interact with pre-existing neurodevelopmental susceptibilities to increase the risk and severity of psychotic symptoms rather than directly altering cortical morphology (Howes and Murray 2014; Niemantsverdriet et al. 2017; Read et al. 2005; Slotema et al. 2012; Varese et al. 2012; Zanarini et al. 1997). Exploratory analyses accounting for trauma-related yielded changes in the observed pattern of findings, with the right TTG effect no longer reaching significance and a new effect emerging in the left opercular part of IFG. These results suggest that trauma-related factors may contribute to the observed structural differences and highlight the challenge of disentangling the respective contributions of AVH and trauma-related psychopathology in BPD. Despite higher rates of PTSD in BPD-AVH group, PTSD diagnosis and CTQ were not associated with AVH severity, suggesting that trauma-related vulnerability may contribute to AVH presence rather than its phenomenological intensity, in line with models proposing partially independent pathways for trauma exposure and symptom expression (Fernyhough 2004; Longden et al. 2012).

From a functional perspective, our findings underscore the importance of early sensory processing abnormalities in the pathogenesis of AVH in BPD. Future studies should aim to integrate structural and functional imaging modalities to investigate whether CG reductions in the rTTG are associated with aberrant functional connectivity patterns, such as hyperactivity in auditory cortices or dysregulated top-down modulation from frontal control regions (Xue et al. 2022). Longitudinal designs will be particularly informative in determining whether such structural alterations precede AVH onset or emerge as a consequence of chronic symptomatology.

The present findings should be interpreted in light of ongoing debates about the nature of AVH in BPD. Although historically framed as “pseudo-psychotic”, growing evidence suggests meaningful overlap with psychotic hallucinations alongside differences in contextual modulation and treatment response (Schroeder et al. 2013; Tseng and Georgiades 2024). Our findings of structural alterations in auditory regions are consistent with the notion that AVH in BPD involve genuine perceptual processes, supporting a dimensional view of hallucinatory phenomena across disorders. The findings may have therapeutic implications for AVH in BPD. Reduced CG in the rTTG suggests that early auditory processing and internal-external source discrimination may represent key intervention targets. Accordingly, neurocognitive, neurofeedback and hallucination-focused cognitive behavioral therapies targeting auditory perception, reality monitoring and misattribution processes have shown promise in reducing hallucination severity and distress (Allen et al. 2008; Linden et al. 2011; Morrison et al. 2014; Slotema et al. 2010). These approaches may complement established first-line treatments of BPD such as Dialectical Behavior Therapy and trauma-informed treatments, offering a more integrated treatment model for the subgroup of patients with BPD who experience AVH.

We acknowledge several potential limitations of this study. First, although our sample size is consistent with prior neuroimaging research in BPD, the fact that the main effect in the rTTG did not survive FDR correction indicates limited statistical power. Accordingly, replication in larger, well-powered samples is essential to exclude false negative and/or false positive findings and robustly determine whether the observed effect reflects a true underlying neurobiological difference. Further, to minimize clinical and phenotypic heterogeneity that previous studies have often attributed to gender (Bozzatello et al. 2024; Sanchious et al. 2024) we only considered female participants. As this study relied on an all-female sample, the generalizability of the findings to male or more diverse populations is limited. Participants with severe substance use disorders were excluded from the sample, but patients with current use of psychotropic drugs fulfilling DSM-5 criteria for moderate substance use disorder (except nicotine) within three months prior to study enrollment were included in the present study. Lifetime substance use histories were not systematically collected, which represents a further limitation of this study. However, meta-analytic neuroimaging studies indicate that structural alterations associated with substance use disorder typically involve widespread prefrontal and limbic regions rather than primary sensory cortical morphology (Pando-Naude et al. 2021; Yan et al. 2023). We, therefore, consider it unlikely that prior substance uses substantially influenced the specific structural measures examined here. Moreover, illness duration (i.e. BPD symptoms) or AVH onset could not be reliably modeled as covariates, as several participants, especially those with childhood-onset voice-hearing, could not recall AVH symptom onset precisely. While age was used as an indirect indicator of BPD illness duration, it should be regarded only as a proxy measure, and its limited precision should be acknowledged. Also, effects of medication cannot be entirely ruled out. However, it is important to note that CG is regarded as a relatively stable early neurodevelopmental marker (Armstrong et al. 1995; White et al. 2010) that is largely established early in life and is not considered highly sensitive to medication effects (Natsuyama et al. 2023; Palaniyappan et al. 2013). Nevertheless, medication exposure may reflect broader aspects of illness severity. In line with this, additional sensitivity analyses of CG (see Supplementary Material) excluding medication covariates resulted in non-significant group differences, suggesting that medication exposure and/or associated clinical severity may contribute to the observed gyrification effects. These findings therefore warrant cautious interpretation. Furthermore, we focused exclusively on CG, GMV and CTh as structural metrics; incorporating additional morphometric indices such as myelination and structural connectivity via diffusion-based techniques could yield a more comprehensive understanding, as alterations in white matter microstructure (e.g., arcuate fasciculus) and demyelination have been repeatedly implicated in the pathophysiology of AVH in SZ (Curcic-Blake et al. 2017; Whitford et al. 2012). Eventually, we infer from cross-sectional data, which clearly imposes limitations on the main phenomenon under study, i.e. AVH, symptoms known to fluctuate in this patient population. A transdiagnostic design combining SZ and BPD could undercover shared AVH neurobiology by distinguishing core neural substrates from disorder specific features, informing targeted interventions (Slotema et al. 2012; Waters 2010). Future studies should adopt longitudinal and multimodal approaches to clarify the temporal dynamics and causal relationships between neurodevelopmental alterations in CG and the emergence of AVH. Investigating how CG reductions in HG interact with functional connectivity and neurotransmitter signaling may further elucidate the pathophysiological underpinnings of AVH and uncover novel therapeutic targets.

In conclusion, this study identified decreased CG in rTTG as a key structural correlate of AVH in BPD, pointing to early auditory processing anomalies as a contributing factor. These results suggest that AVH in BPD may be linked to prenatal neurodevelopmental alterations in the TTG, highlighting the role of early auditory processing vulnerabilities rather than the frontal source-monitoring deficits more commonly described in SZ. While AVH in BPD remain incompletely understood, our findings point to the importance of considering early sensory anomalies in their pathogenesis, with potential therapeutic implications for interventions targeting internal-external sound source discrimination.

## Supplementary Information

Below is the link to the electronic supplementary material.

Supplementary Material 1

## Data Availability

Data will be made available on scientifically reasonable requests.
