# Supplementary material for: Structural alterations in early auditory cortex in borderline personality disorder with auditory verbal hallucinations
Source: Brain Struct Funct. 2026 Aug 29;231(7):127. doi: 10.1007/s00429-026-03162-0 (PMC13526128; doi:10.1007/s00429-026-03162-0)
Supplement: Supplementary file 1 [file 429_2026_3162_MOESM1_ESM.docx]

**Supplementary material**

*Detailed TASH processing pipeline*

T1-weighted anatomical MRI scans were processed using the FreeSurfer software suite (v. 7.4.1) on an Ubuntu 22.04 LTS system, with data analyzed within a “tcsh” shell environment to ensure compatibility with the TASH toolbox scripts. The initial anatomical processing involved FreeSurfer’s standard recon-all pipeline, which performs skull stripping, Talairach transformation, intensity normalization, cortical surface tessellation and inflation, and anatomical labeling.

The TASH toolbox, originally developed for FreeSurfer version 5.3, required modifications to be compatible with FreeSurfer 7.4.1. First, in the script TASH_Measures.m, the number of header lines skipped when reading FreeSurfer’s *.stats files was increased from 53 to 61 (at lines 12 and 32) to accommodate changes in the file format introduced in newer FreeSurfer versions. Additionally, the subject definition files TASH_DefineSubjects.m and TASH_subjects.m were edited to correctly specify participant IDs and to accurately point to the FreeSurfer subjects directory where preprocessed data were stored. To match the syntax requirements of the TASH shell scripts, the working shell environment was switched to tcsh, as the default bash shell is incompatible with these scripts. MATLAB (v. R2024a) was verified to be accessible and callable directly from the terminal, a necessary requirement for running TASH’s MATLAB-based processing steps.

The TASH processing pipeline output included detailed anatomical segmentations of Heschl’s gyrus, stored in subject-specific subdirectories within FreeSurfer’s subjects folder. These outputs consisted of volumetric files and surface patch definitions in newly created folders such as RestrictedFlatPatches and Volume. Screenshots of the segmentation results generated via FreeSurfer’s visualization tools provided an additional layer of quality control.

All environment variables, including SUBJECTS_DIR and FREESURFER_HOME, were appropriately set and sourced in the tcsh shell to ensure proper script execution. The TASH pipeline was executed using the source TASH.csh command within the working directory containing the TASH toolbox files.

*Non-significant between-group differences*

**Right Hemisphere; Gray Matter Volume**

Middle Temporal Gyrus F (2, 71) = 57.55, p = 0.69

Planum Temporal F (2, 71) = 2.97, p = 0.66

Superior Temporal Gyrus F (2, 71) = 25.86, p = 0.91

Transverse Temporal Gyrus F (2, 71) = 1.3, p = 0.48

Triangular part of Inferior Frontal Gyrus F (2, 71) = 7.87, p = 0.18

Opercular part of the Inferior Frontal Gyrus BPD vs. AVH: t = 0.11, p = 0.91; HC vs. AVH: t = 1.30, p = 0.20

Orbital part of Inferior Frontal Gyrus F (2, 71) = 1.77, p = 0.11

**Right Hemisphere; Cortical Thickness**

Middle Temporal Gyrus F (2, 72) = 1.30, p = 0.62

Planum Temporal F (2, 72) = 1.24, p = 0.90

Superior Temporal Gyrus F (2, 72) = 1.42, p = 0.19

Transverse Temporal Gyrus BPD vs. AVH: t = -1.62, p = 0.11 ; HC vs. AVH : t = -1.1, p = 0.29

Triangular part of Inferior Frontal Gyrus F (2, 72) = 1.25, p = 0.15

Opercular part of the Inferior Frontal Gyrus BPD vs. AVH: t = -1.82, p = 0.07; HC vs. AVH: t = -0.52, p = 0.60

Orbital part of Inferior Frontal Gyrus F (2, 72) = 1.74, p = 0.40

**Right Hemisphere; Cortical Gyrification**

Middle Temporal Gyrus F (2, 72) = 68.41, p = 0.94

Planum Temporal F (2, 72) = 91.87, p = 0.55

Superior Temporal Gyrus F (2, 72) = 67.70, p = 0.63

Triangular part of Inferior Frontal Gyrus BPD vs. AVH: t = 0.72, p = 0.47; HC vs. AVH: t = 0.20, p = 0.84

Opercular part of the Inferior Frontal Gyrus F (2, 72) = 81.55, p = 0.90

Orbital part of Inferior Frontal Gyrus F (2, 72) = 103.03, p = 0.72

**Left Hemisphere; Gray Matter Volume**

Middle Temporal Gyrus F (2, 71) = 64.78, p = 0.83

Planum Temporal F (2, 71) = 3.68, p = 0.64

Superior Temporal Gyrus F (2, 71) = 23.12, p = 0.87

Transverse Temporal Gyrus F (2, 71) = 1.9, p = 0.98

Triangular part of Inferior Frontal Gyrus F (2, 71) = 11.29, p = 0.52

Opercular part of the Inferior Frontal Gyrus BPD vs. AVH: t = -0.70, p = 0.50; HC vs. AVH: t = 0.50, p = 0.62

Orbital part of Inferior Frontal Gyrus F (2, 71) = 1.77, p = 0.91

**Left Hemisphere; Cortical Thickness**

Middle Temporal Gyrus F (2, 72) = 1.42, p = 0.59

Planum Temporal F (2, 72) = 1.16, p = 0.54

Superior Temporal Gyrus F (2, 72) = 1.2, p = 0.81

Transverse Temporal Gyrus F (2, 72) = 2.22, p = 0.56

Triangular part of Inferior Frontal Gyrus BPD vs. AVH: t = -1.20, p = 0.25; HC vs. AVH: t = -1.44, p = 0.15

Opercular part of the Inferior Frontal Gyrus BPD vs. AVH: t = -1.11, p = 0.27; HC vs. AVH: t = -1.63, p = 0.11

Orbital part of Inferior Frontal Gyrus BPD vs. AVH: t = -0.93, p = 0.35; HC vs. AVH: t = -1, p = 0.34

**Left Hemisphere; Cortical Gyrification**

Middle Temporal Gyrus F (2, 72) = 87.97, p = 0.68

Planum Temporal F (2, 72) = 64.98, p = 0.12

Superior Temporal Gyrus F (2, 72) = 77.1, p = 0.83

Transverse Temporal Gyrus F (2, 72) = 406.53, p = 0.07

Triangular part of Inferior Frontal Gyrus F (2, 72) = 119.71, p = 0.92

Opercular part of the Inferior Frontal Gyrus F (2, 72) = 96.7, p = 0.11

Orbital part of Inferior Frontal Gyrus F (2, 72) = 130.13, p = 0.11

*Model CG without correction for medication*

**Right Hemisphere**

Middle Temporal Gyrus F (2, 73) = 68.49, p = 0.96

Planum Temporal F (2, 73) = 92.46, p = 0.45

Superior Temporal Gyrus F (2, 73) = 69.33, p = 0.28

Transverse Temporal Gyrus F (2, 73) = 416.44, p = 0.058

Triangular part of Inferior Frontal Gyrus BPD vs. AVH: t = 0.75, p = 0.46; HC vs. AVH: t = 0.16, p = 0.87

Opercular part of the Inferior Frontal Gyrus F (2, 73) = 83.19, p = 0.69

Orbital part of Inferior Frontal Gyrus F (2, 73) = 104.61, p = 0.91

**Left Hemisphere**

Middle Temporal Gyrus F (2, 73) = 88.20, p = 0.69

Planum Temporal F (2, 73) = 67.31, p = 0.15

Superior Temporal Gyrus F (2, 73) = 78.46, p = 0.61

Transverse Temporal Gyrus F (2, 73) = 421.82, p = 0.18

Triangular part of Inferior Frontal Gyrus F (2, 73) = 119.93, p = 0.96

Opercular part of the Inferior Frontal Gyrus F (2, 73) = 96.72, p = 0.07

Orbital part of Inferior Frontal Gyrus F (2, 73) = 136.17, p = 0.34

*Model CG corrected for depression*

**Right Hemisphere**

Middle Temporal Gyrus F (2, 71) = 68.39, p = 0.93

Planum Temporal F (2, 71) = 89.78, p = 0.29

Superior Temporal Gyrus F (2, 71) = 67.51, p = 0.60

Triangular part of Inferior Frontal Gyrus BPD vs. AVH: t = 0.37, p = 0.71; HC vs. AVH: t = -0.59, p = 0.56

Opercular part of the Inferior Frontal Gyrus F (2, 71) = 81.53, p = 0.90

Orbital part of Inferior Frontal Gyrus F (2, 71) = 102.96, p = 0.76

**Left Hemisphere**

Middle Temporal Gyrus F (2, 71) = 86.78, p = 0.73

Planum Temporal F (2, 71) = 66.62, p = 0.15

Superior Temporal Gyrus F (2, 71) = 76.80, p = 0.78

Transverse Temporal Gyrus F (2, 71) = 405.45, p = 0.13

Triangular part of Inferior Frontal Gyrus F (2, 71) = 119.63, p = 0.91

Opercular part of the Inferior Frontal Gyrus F (2, 71) = 95.81, p = 0.11

Orbital part of Inferior Frontal Gyrus F (2, 71) = 129.99, p = 0.18

*Model CG with CTQ as covariate*

**Right Hemisphere**

Middle Temporal Gyrus F (2, 71) = 68.22, p = 0.93

Planum Temporal F (2, 71) = 88.94, p = 0.67

Superior Temporal Gyrus F (2, 71) = 67.05, p = 0.89

Transverse Temporal Gyrus F (2, 71) = 396.85, p = 0.07

Triangular part of Inferior Frontal Gyrus BPD vs. AVH: t = 0.27, p = 0.79; HC vs. AVH: t = -0.53, p = 0.60

Opercular part of the Inferior Frontal Gyrus F (2, 71) = 81.50, p = 0.89

Orbital part of Inferior Frontal Gyrus F (2, 71) = 102.99, p = 0.88

**Left Hemisphere**

Middle Temporal Gyrus F (2, 71) = 86.93, p = 0.45

Planum Temporal F (2, 71) = 66.31, p = 0.14

Superior Temporal Gyrus F (2, 71) = 76.65, p = 0.75

Transverse Temporal Gyrus F (2, 71) = 406.28, p = 0.40

Triangular part of Inferior Frontal Gyrus F (2, 71) = 119.50, p = 0.89

Opercular part of the Inferior Frontal Gyrus F (2, 71) = 88.90, p = 0.007

Orbital part of Inferior Frontal Gyrus F (2, 71) = 128.16, p = 0.17

*Model CG with PTSD as covariate*

**Right Hemisphere**

Middle Temporal Gyrus F (2, 71) = 67.46, p = 0.80

Planum Temporal F (2, 71) = 89.16, p = 0.63

Superior Temporal Gyrus F (2, 71) = 67.35, p = 0.53

Transverse Temporal Gyrus F (2, 71) = 404.60, p = 0.07

Triangular part of Inferior Frontal Gyrus BPD vs. AVH: t = 0.95, p = 0.35; HC vs. AVH: t = 0.40, p = 0.69

Opercular part of the Inferior Frontal Gyrus F (2, 71) = 81.43, p = 0.85

Orbital part of Inferior Frontal Gyrus F (2, 71) = 101.16, p = 0.86

**Left Hemisphere**

Middle Temporal Gyrus F (2, 71) = 87.36, p = 0.91

Planum Temporal F (2, 71) = 65.31, p = 0.42

Superior Temporal Gyrus F (2, 71) = 76.71, p = 0.87

Transverse Temporal Gyrus F (2, 71) = 405.53, p = 0.18

Triangular part of Inferior Frontal Gyrus F (2, 71) = 116.85, p = 0.80

Opercular part of the Inferior Frontal Gyrus F (2, 71) = 96.10, p = 0.11

Orbital part of Inferior Frontal Gyrus F (2, 71) = 128.78, p = 0.17

*Model CG with CTQ and PTSD as covariates*

**Right Hemisphere**

Middle Temporal Gyrus F (2, 70) = 67.45, p = 0.81

Planum Temporal F (2, 70) = 88.59, p = 0.86

Superior Temporal Gyrus F (2, 70) = 66.12, p = 0.69

Transverse Temporal Gyrus F (2, 70) = 393.63, p = 0.06

Triangular part of Inferior Frontal Gyrus BPD vs. AVH: t = 0.63, p = 0.53; HC vs. AVH: t = -0.94, p = 0.35

Opercular part of the Inferior Frontal Gyrus F (2, 70) = 81.42, p = 0.86

Orbital part of Inferior Frontal Gyrus F (2, 70) = 100.59, p = 0.71

**Left Hemisphere**

Middle Temporal Gyrus F (2, 70) = 85.35, p = 0.51

Planum Temporal F (2, 70) = 65.28, p = 0.45

Superior Temporal Gyrus F (2, 70) = 75.75, p = 0.81

Transverse Temporal Gyrus F (2, 70) = 404.65, p = 0.43

Triangular part of Inferior Frontal Gyrus F (2, 70) = 115.46, p = 0.58

Opercular part of the Inferior Frontal Gyrus F (2, 70) = 88.82, p = 0.007

Orbital part of Inferior Frontal Gyrus F (2, 70) = 124.85, p = 0.09

**Supplemental figures**

**Supplemental figure S1**: Visualization of the masks used for region of interest-based analyses (left); middle temporal gyrus (light blue), opercular part of the inferior frontal gyrus (yellow), orbital part of the inferior frontal gyrus (green), planum temporal (dark blue), superior temporal gyrus (dark red), triangular part of the inferior frontal gyrus (beige) and transverse temporal gyrus (light red). Visualization of right transverse temporal gyrus (right). Masks created with MarsBaR (<https://marsbar-toolbox.github.io/download.html> last accessed: September 19, 2025) and displayed as an overlay onto the “mni152” template implemented in MRIcroGL. This figure was created with MRIcroGL (https://www.nitrc.org/projects/mricrogllast accessed: September 19, 2025).

**Supplemental figure S2**: Visualization of rTTG. Masks created with MarsBaR (<https://marsbar-toolbox.github.io/download.html> last accessed: September 19, 2025) and displayed as an overlay onto the “mni152” template implemented in MRIcroGL. This figure was created with MRIcroGL (https://www.nitrc.org/projects/mricrogllast accessed: March 11, 2026).

**Supplemental figure S3**: Visualization of CTQ distributions over groups. Figure created with R (https://www.r-project.org/ last accessed: February 16, 2026).
